# Effects of Including Sprints in One Weekly Low-Intensity Training Session During the Transition Period of Elite Cyclists

**DOI:** 10.3389/fphys.2020.01000

**Published:** 2020-09-11

**Authors:** Nicki Winfield Almquist, Ine Løvlien, Per Thomas Byrkjedal, Matt Spencer, Morten Kristoffersen, Knut Skovereng, Øyvind Sandbakk, Bent R. Rønnestad

**Affiliations:** ^1^Section for Health and Exercise Physiology, Inland Norway University of Applied Sciences, Lillehammer, Norway; ^2^Centre for Elite Sports Research, Department of Neuromedicine and Movement Science, Norwegian University of Science and Technology, Trondheim, Norway; ^3^Department of Sport Science and Physical Education, University of Agder, Kristiansand, Norway; ^4^Department of Sport, Food and Natural Sciences, Western Norway University of Applied Sciences, Bergen, Norway

**Keywords:** periodization strategies, off-season, sprint training, elite athletes, athlete burnout questionnaire

## Abstract

The purpose of this study was to investigate the effects of including 30-s sprints in one weekly low-intensity training (LIT) session during a 3-week transition period in elite cyclists. Sixteen male elite cyclists (maximal oxygen uptake, VO_2max_: 72 ± 5 ml·kg^−1^·min^−1^) reduced their training load by ~60% for 3 weeks from the end of competitive season and performed only LIT or included 30-s sprints (SPR) in one weekly LIT-session. Performance and physiological capacities were evaluated during a prolonged (~2.5 h) test-session, including a strength test, a submaximal blood lactate profile test, an incremental test to exhaustion to determine VO_2max_, 1 h continuous cycling including four maximal 30-s sprints, and a 20-min all-out test. In addition, mental recovery was evaluated using the Athlete Burnout Questionnaire (ARQ). The only significant between-group change during the transition period was an 8 ± 11% larger improvement in 30-s sprint performance in SPR compared to control (CON; SPR: 4 ± 5%, CON: −4 ± 5%, *p* = 0.01). Although not different from CON, SPR maintained 20-min all-out performance (−1 ± 5%, *p* = 0.37) and fractional utilization of VO_2max_ (1.9 ± 6.1%-points, *p* = 0.18) during the 20-min all-out test, whereas corresponding declines were observed in CON (−3 ± 5%, *p* = 0.04, and −2.5 ± 2.9%-points, *p* = 0.02, respectively). Power output at 4 mmol·L^−1^ blood lactate concentration decreased similarly in SPR (−4 ± 4%, *p* = 0.02) and CON (−5 ± 5%, *p* = 0.01), while VO_2max_, maximal aerobic power (W_max_), and total burnout score were unaffected in both groups. Including sprints in one weekly LIT-session in the transition period improves sprint performance and maintains 20-min all-out power and fractional utilization of VO_2max_ without compromising mental recovery. Inclusion of sprints in LIT-sessions may therefore be a plausible, time-efficient strategy during short periods of reduced training.

## Introduction

The annual training season for an elite cyclist can be broken into three distinct periods, the preparatory, competition, and transition period (Mujika et al., 2018). Elite cyclists typically spend up to 100 days in competition (Lucia et al., 2001), which is both a high physical and psychological exertion, with an inherent risk of burnout toward the end of the season (Silva, 1990; Lemyre et al., 2006). Although the need for a subsequent period of physical and mental recovery is regarded as necessary for elite athletes (Mujika et al., 2018), the manipulation of training in these transition periods is scarcely investigated (Garcia-Pallares et al., 2009; Ronnestad et al., 2014). To recover from the strenuous competition period, cyclists’ training load is often drastically reduced for 2–3 weeks in the subsequent transition period (Lucia et al., 2000; Sassi et al., 2008). However, too long periods (>4 weeks) of training cessation might lead to deterioration of performance (Mujika and Padilla, 2000; Decroix et al., 2016; Maldonado-Martin et al., 2017).

Maintaining a minimum of training load in periods of decreased training volume seems necessary to avoid performance decrements (Mujika, 1998; Bosquet et al., 2007), with high-intensity training (HIT) playing a key role for maintenance of endurance performance (Neufer, 1989; Garcia-Pallares et al., 2009; Ronnestad et al., 2014). Maintenance of fitness in the transition period might also be crucial for continuous improvement in the following seasons of elite athletes (Mujika et al., 1995). Indeed, a study by Ronnestad et al. (2014) on well-trained cyclists showed that performing a HIT session every 7–10 days during an 8-week period following the competition period maintained power output at 4 mmol·L^−1^ [BLa^−^], maximal oxygen uptake (VO_2max_), and 40-min all-out performance better than low-intensity training (LIT; Ronnestad et al., 2014). However, performing HIT-sessions during the transition period where physical and mental recovery is needed might be too strenuous, leading to overreaching and burnout. Therefore, including sprint (SPR) training instead might be a beneficial, low-load alternative for elite cyclists.

Short maximal-effort intervals have been reported to be of less strain compared to longer HIT-intervals (Valstad et al., 2018) and might serve as an intensive stimulus, sufficient for maintaining endurance performance in shorter periods of reduced training volume. For example, the addition of sprint training in periods with 25–65% reductions in training volume has shown to maintain endurance performance-determining factors in moderately trained athletes (VO_2max_, muscle oxidative capacity, and capillarization; Joyner and Coyle, 2008) and improved performance at or above intensities eliciting VO_2max_ (Bangsbo et al., 2009; Iaia et al., 2009; Skovgaard et al., 2018). Furthermore, including 30-s sprints every 10 min in 60-min LIT-sessions during an 8-week intervention has recently shown improved performance in trained cyclists (Gunnarsson et al., 2019). Therefore, implementing 30-s sprints in habitual LIT-sessions for short transition periods (3 weeks) might be a time-efficient strategy of relatively low strain for maintaining endurance performance.

Therefore, the main aim of this study was to investigate the effect of including 30-s sprints in one weekly LIT-session during a 3-week transition period on measures of sprint and endurance performance in elite cyclists, as well as the associated changes in physiological capacities and mental recovery. We hypothesized that inclusion of sprints during the transition period would improve sprint performance and maintain endurance performance-related measures compared to LIT only.

## Materials and Methods

### Participants and Ethics Statement

Twenty-one cyclists volunteered for the study. Two participants withdrew due to circumstances unrelated to the study and three participants were excluded due to sickness or lack of adherence to the intervention, leaving a total of 16 participants. Physiological parameters, participants’ characteristics, and training volume are presented in Table 1. All participants were informed of the possible risks and discomforts associated with the study and all gave their written informed consent to participate before commencing the study. The study was approved by the Local Ethical Committee at Inland Norway University of Applied Sciences and performed according to the Declaration of Helsinki, 1975. The study was a multi-center study conducted at four Norwegian universities with identical laboratory equipment using the same standardized testing procedures supervised by the same physician. To categorize the cyclists, the physiological characteristics suggested by De Pauw et al. (2013) was used. Eleven participants were regarded as performance level 5 athletes (VO_2max_: >71 ml·kg^−1^·min^−1^, W_max_: >5.5 W·kg^−1^) and five participants were regarded as level 4 athletes (VO_2max_: 65–71 ml·kg^−1^·min^−1^, W_max_: 4.9–6.4 W·kg^−1^), hence referred to as elite cyclists.

**Table 1:** Participants’ characteristics measured 3–5 days after each cyclists’ last competition and weekly training volume in the last 4 weeks of the competition period.

	SPR *n* = 7	CON *n* = 9	Group diff.
Age (years)	22.9 ± 3.0	21.1 ± 3.9	*p* = 0.32
Body mass (kg)	73.6 ± 9.0	73.1 ± 4.8	*p* = 0.89
VO_2max_ (L·min^−1^)	5.4 ± 0.7	5.2 ± 0.5	*p* = 0.57
VO_2max_ (ml·kg^−1^·min^−1^)	73.4 ± 4.9	71.3 ± 4.5	*p* = 0.40
W_max_ (W)	439 ± 58	442 ± 48	*p* = 0.93
Power output at 4 mmol·L^−1^ [BLa^−^] (W)	328 ± 66	321 ± 41	*p* = 0.80
Training volume 30 days prior to inclusion (h·wk.^−1^)	14 ± 4	12 ± 3	*p* = 0.33
Reduction in iTRIMP training load (%)	−62 ± 9	−64 ± 11	*p* = 0.72

VO_2max_, maximal oxygen uptake; W_max_, maximal minute power output (W), reduction in training load per week from 4 week of competition period to 3 week of transition period quantified using individualized TRIMP, mean ± SD and matching of groups.

### Experimental Design

The intervention was initiated 3–5 days after each cyclist’s last competition of the season and was carried out over 21.2 ± 0.4 days. The participants were randomly assigned to either a SPR group or a control (CON) group. During the 4 weeks prior to the intervention, the cyclists performed on average the same number of training sessions per week (SPR: 6.4 ± 0.7 vs. CON: 6.2 ± 1.1 sessions, *p* = 0.80) of which an equal amount was characterized as HIT-sessions (SPR: 15 ± 10% vs. CON: 15 ± 9%, *p* = 0.95) and the training load from HIT was not different between groups (*p* = 0.24). SPR and CON reduced training load from the competition period to the transition period equally (Table 1), and only LIT was performed during the intervention (SPR: 13 ± 4 vs. CON: 12 ± 3 sessions, *p* = 0.58). However, once a week SPR performed a supervised 90-min LIT-session, riding at a power output equivalent to 60% of VO_2max_, including three sets of 3 × 30-s maximal sprints, interspersed by 4-min of active recovery (100 W) and 15 min between sets. CON performed a time-matched supervised session at a power output equivalent to 60% of VO_2max_.

### Testing Procedures

**Figure 1:**
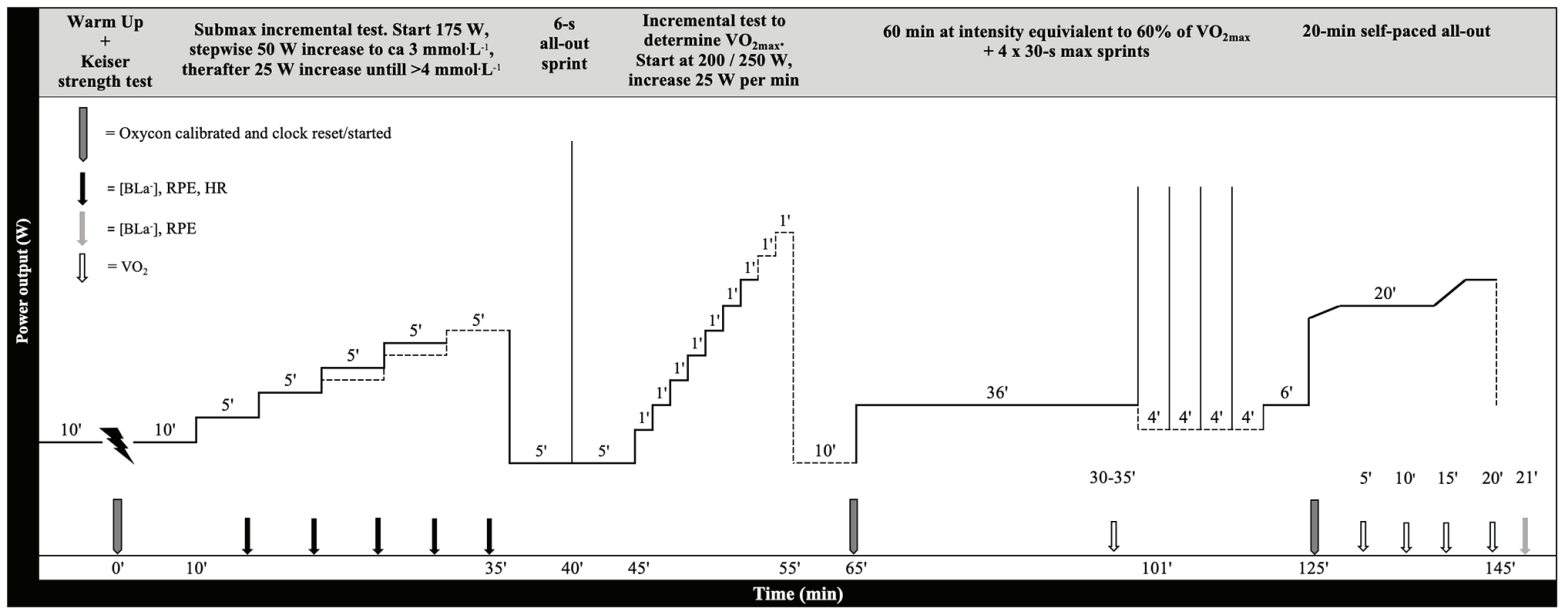
Schematic illustration of the test protocol, including strength test, blood lactate [BLa^−^] profile, 6-s all-out sprint, incremental test to exhaustion, and 60 min continuous cycling including 4 × 30-s maximal sprints and 20-min all-out.

The participants were instructed to refrain from caffeine, beta-alanine, and bicarbonate 24 h prior to testing. Participants were also instructed to register and duplicate food intake and time of consumption 24 h prior to both tests, but food diaries were not collected. All testing was performed on the same time of the day (±1 h) in a controlled environmental condition (16–18°C and 20–35% relative humidity) with a fan ensuring air circulation around the rider. A schematic presentation of the prolonged test protocol is outlined in Figure 1.

### Strength Test

After a 10-min cycling warm-up at self-selected power output (150–200 W) a predetermined, standardized, 10-repetition incremental leg press test set to 250 kg for all participants on a Keiser AIR300 horizontal leg-press dynamometer (Keiser Sport health equipment INC., Fresno, CA) was initiated. Changes in strength parameters might affect the sprint ability and was therefore included (Ronnestad et al., 2017). The Keiser AIR300 uses pneumatic resistance to measure force and velocity in each repetition. The incremental test was performed in the seated position with a 90° knee-joint angle, starting at 41 kg and increasing to 250 kg at the tenth repetition with increased and standardized increments and rest-periods between repetitions. If the participant exceeded 250 kg, the test continued with 60-s rest between attempts until failure. The participants were instructed to push as explosively as possible until failure. The theoretical, maximal velocity (V_max_), maximal force (F_max_), and maximal power (P_max_) was then calculated based on the second-order polynomial relationship between force and power (Colyer et al., 2018).

### Blood Lactate Profile

After a 5-min break, a blood lactate [BLa^−^] profile test to determine the relationship between power output, and [BLa^−^] concentration during a submaximal continuous incremental test was initiated. This test has previously been described in detail (Ronnestad et al., 2010). Briefly, participants cycled for 5 min at 175 W, followed by 50-W increments every 5 min until a [BLa^−^] of 3 mmol·L^−1^, after which increments were 25 W. The test was terminated at a [BLa^−^] of 4 mmol·L^−1^ or higher. All cycling tests were performed on an electromagnetic braked cycle ergometer (Lode Excalibur Sport, Lode B. V., Groningen, The Netherlands), which was adjusted to each cyclists’ individual preferences and replicated throughout all testing. The fixed modus was used during continuous cycling, allowing the cyclists to freely choose frequency with a fixed resistance. VO_2_ measurements started from 2 min into every bout and VO_2_ was calculated as an average from 2.5 to 4.5 min. VO_2_ was measured using a computerized metabolic system with mixing chamber (Oxycon Pro, Erich Jaeger, Hoechberg, Germany), which was calibrated every hour. Blood was sampled from the fingertip on completion of each 5-min bout and analyzed for whole blood [BLa^−^] using a lactate analyzer (Biosen C line, EKF Diagnostic, Germany). Heart rate (HR) was recorded at the end of each steady-state increment using the participants’ own HR-monitor and rate of perceived exertion (RPE) was recorded according to Borg Scale 6–20. Based on these measures, the power output at 4 mmol·L^−1^ [BLa^−^] was calculated by interpolation and was used as a submaximal performance measure to compare each participant from Pre to Post.

### 6-s All-Out Sprint

After 5 min of active recovery, a 6-s all-out sprint was performed in the seated position with a stationary start and a resistance of 0.8 Nm∙kg^−1^ body mass. Peak power output was defined as the highest value achieved during the 6-s all-out with recordings at 6 Hz.

### VO_2max_ Test

After an additional 5 min of active recovery at ~150 W, an incremental test to exhaustion to determine VO_2max_ was initiated at 200 or 250 W depending on previous individual results. Power output increased by 25 W every minute until the RPM decreased below 60 min^−1^ despite audible encouragement from the test leader. VO_2max_ was calculated as the highest average of a 1-min moving average using 5-s VO_2_-measurements and peak heart rate (HR_peak_) was registered. W_max_ was calculated as the mean power output during the last minute of the incremental test.

### 60 min Continuous Cycling With 4 × 30-s Maximal Sprints and Subsequently 20-min All-Out

Ten minutes after the incremental test to exhaustion, the participants proceeded with a 60-min continuous cycling session at an intensity equivalent to 60% of VO_2max_, which was calculated from the [BLa-] profile and VO_2max_ using interpolation. Four repeated 30-s maximal sprints separated by 4 min active recovery (100 W) were undertaken between 36–50 min and the test was concluded by a self-paced 20-min all-out without rest-periods in between (Figure 1). The chosen intensity of 60% of VO_2max_ corroborates well with reported intensities of competitions (van Erp and Sanders, 2020), making the repeated sprints and 20-min all-out competition-relevant performance measures. At Post, the participants rode at the same power output as Pre during the 60-min continuous cycling. The start power output on the 20-min all-out was self-selected at Pre and power and cadence was self-administered throughout the Pre and Post tests, however, the participants were blinded to the average power output. The start power output was replicated at Post to ensure the same pacing conditions. VO_2_, HR, RPE, and [BLa^−^] were measured during the test, according to Figure 1. During sprints, the resistance was set to 0.8 Nm·kg^−1^ in the Wingate modus and started at 80 RPM. Mean power output was presented as the 30-s average power output sustained throughout each maximal 30-s sprint. Fractional utilization of VO_2max_ during the 20-min all-out was calculated from an average of respiratory VO_2_-measurements obtained in the periods 4–5, 9–10, 14–15, and 19–20 min, expressed relatively to VO_2max_ obtained at the respective time-point. VO_2_-measurements started 30-s prior to each period to ensure steady measures of VO_2_. Water, energy-drink in standard mixture according to manufacturer’s description (HIGH-5, UK), and gels (SIS Isotonic Energy Gel, UK) without caffeine were provided ad libitum after the incremental test to exhaustion and throughout the test. All participants but one ingested energy-drink and gels during the experimental tests. The amount was recorded and repeated at Post to ensure the same relative hydration-level. On average, 745 ± 369 ml energy-drink and 44 ± 21 ml gel were consumed at Pre and 811 ± 454 ml (*p* = 0.37) energy-drink and 38 ± 24 ml gel (*p* = 0.17) were consumed at Post.

Gross efficiency (GE) was defined as the ratio between the mechanical power output (PO) and the metabolic power input (PI) calculated using VO_2_ measurements and the energetic equivalent (Peronnet and Massicotte, 1991) PI = VO_2_ L·s^−1^ x (4,840 J·L^−1^ × RER + 16,890 J·L^−1^). GE was calculated from the [BLa-] profile test in the fresh state using the power output closest to that each participant rode at in the 60-min continuous cycling test. Equivalently, the GE in the semi-fatigued state was calculated using the steady-state period before sprinting (30–35 min) in the 60-min continuous cycling test (Figure 1). The power output was not different in fresh and semi-fatigued state in SPR (fresh: 227 ± 39 vs. semi-fatigued: 225 ± 41 W, *p* = 0.71) or CON (fresh: 215 ± 28 W vs. semi-fatigued: 219 ± 30 W, *p* = 0.35).

### Training Load and Administration

**Figure 2:**
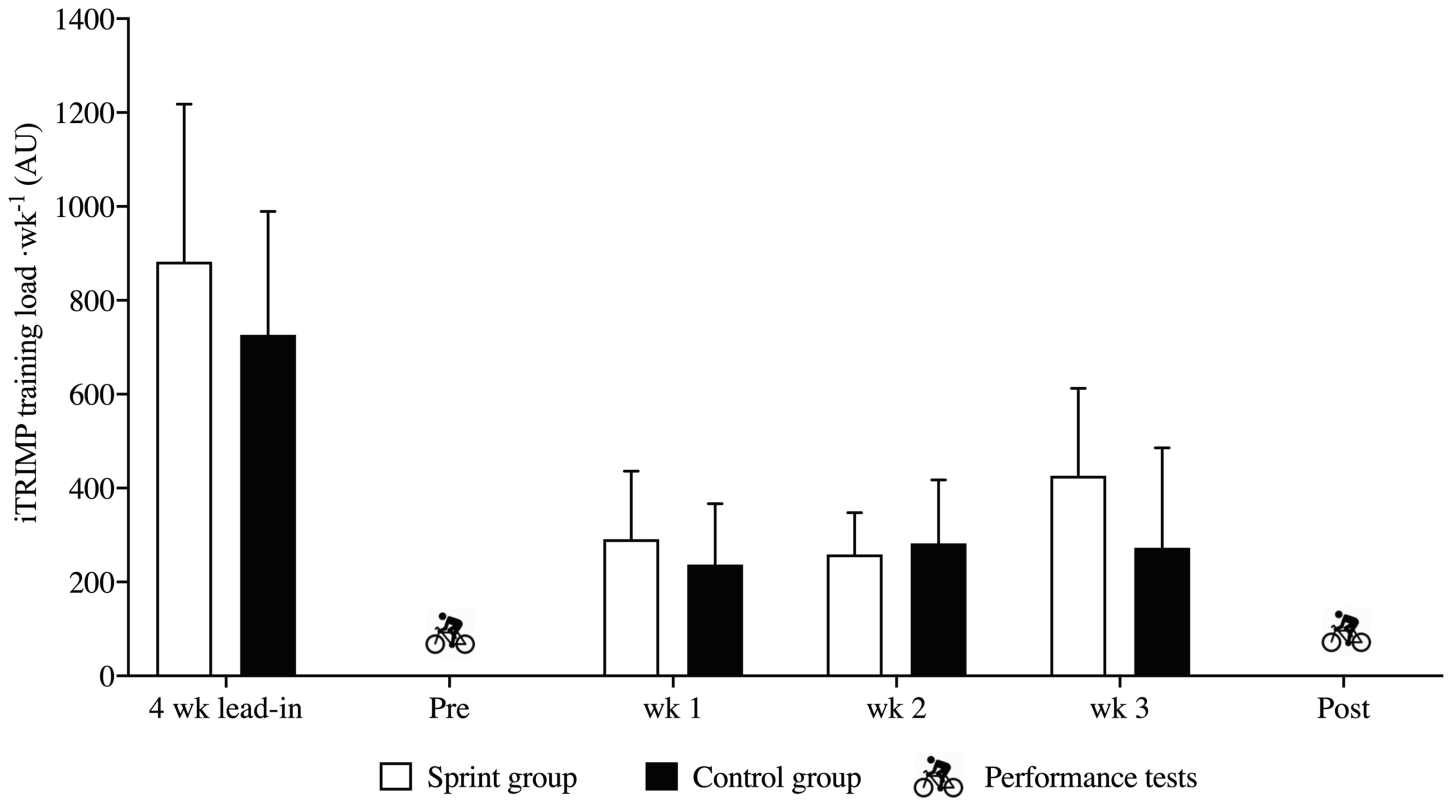
Training load during lead-in and 3 week intervention using the individualized TRIMP method. Mean ± SD.

Training load was quantified using the individualized training impulse (iTRIMP) as described elsewhere (Manzi et al., 2009), by weighing exercise intensity according to an individual’s own HR vs. [BLa^−^] relationship, calculated by line of best fit from the lactate profile and VO_2max_ test. iTRIMP uses the weighting factor y_i_, which increases exponentially based on the HR vs. [La^−^] relationship to weight every HR. An accumulated iTRIMP score was calculated by the following equation:

$$\mathrm{iTRIMP}\;\left(\mathrm{arbitrary units}\;\left(\mathrm{AU}\right)\right)=D\;\left(\min\right)\;x\;\Delta HR_{\mathrm{ratio}}\;x\;y_{i}$$

where ΔHR_ratio_ is calculated from (HR_work_−HR_rest_)/(HR_max_−HR_rest_), and D is time spent exercising. Design and training load administration is specified in Figure 2.

### Athlete Burnout Questionnaire

To evaluate mental recovery, the 15-item sport-specific Athlete Burnout Questionnaire (ABQ) was used (Raedeke and Smith, 2001). Athletes were asked to rate “How often do you feel this way?” in 15 different statements to evaluate their participation motives in their sport on a 5-point Likert-scale from 1 = almost never to 5 = almost always. The ABQ has three 5-item subscales assessing three key dimensions of burnout: (1) reduced sense of accomplishment (e.g., “It seems that no matter what I do, I don’t perform as well as I should”), (2) emotional and physical exhaustion (e.g., “I feel so tired from my training that I have trouble finding energy to do other things”), and (3) devaluation of sport participation (e.g., “The effort I spend participating in my sport would be better spent doing other things”). A total summarized score for the ABQ is achieved by averaging all three subscale scores. The questionnaires were completed at Pre and Post.

### Statistics

Variables were tested for normal distribution using Shapiro-Wilk test. Based on a study on amateur road cyclists performing sprint training (Fortes et al., 2019), power calculations were made to determine the minimum of participants to include in the present study to detect changes in sprint performance. Based on the estimated effect size (ES) of 0.60 in changes in sprint performance when reducing training load for 3 weeks (Fortes et al., 2019) together with an alpha-level of 0.05, a power of 0.80, and a correlation between repeated measures of 0.50, the minimum sample size needed to determine significant differences in sprint performance was calculated to be eight in each group. A mixed linear model was applied to compare relative changes between groups in physiological, performance, and strength measures with group (and sprint) defined as fixed effects and corrected using Pre-values as a covariate using the software SPSS v.25. To compare main effects of time, a mixed linear model was applied with fixed effects defined by group, and time and random effects were defined by subject. Data are presented as mean ± SD. To evaluate the relationship between percentage changes in 20-min all-out performance and other performance measures, a stepwise, multiple linear regression was applied. The percentage changes in power output at 4 mmol·L^−1^ [BLa^−^], absolute VO_2max_, W_max_, [BLa^−^], and RPE at the end of 20-min all-out and fractional utilization during 20-min all-out, 30-s sprint performance, and GE in the semi-fatigued state were included in the model. For values expressed in %, the changes were calculated as percentage-points (%-points) by subtracting Post-values from Pre-values. All variables included in the final model had a variance inflation factor between 1.2–1.6 and *p* < 0.05. Whenever a significant main effect was obtained, a Sidak *post hoc* analysis was performed with an alpha-level of 0.05. Values of *p* > 0.05 and *p* < 0.1 were described as approaching significance. Hopkins’ ES using pooled SD ± 95% confidence interval (CI) was calculated to highlight the practical significance of differences in performance changes between groups. Interpretations of the magnitude of ES were as follows: <0.2 trivial, 0.2–0.6 small, 0.6–1.2 moderate, 1.2–2.0 large, and 2.0–4.0 very large difference (Hopkins et al., 2009).

## Results

### Sprint Performance

**Figure 3:**
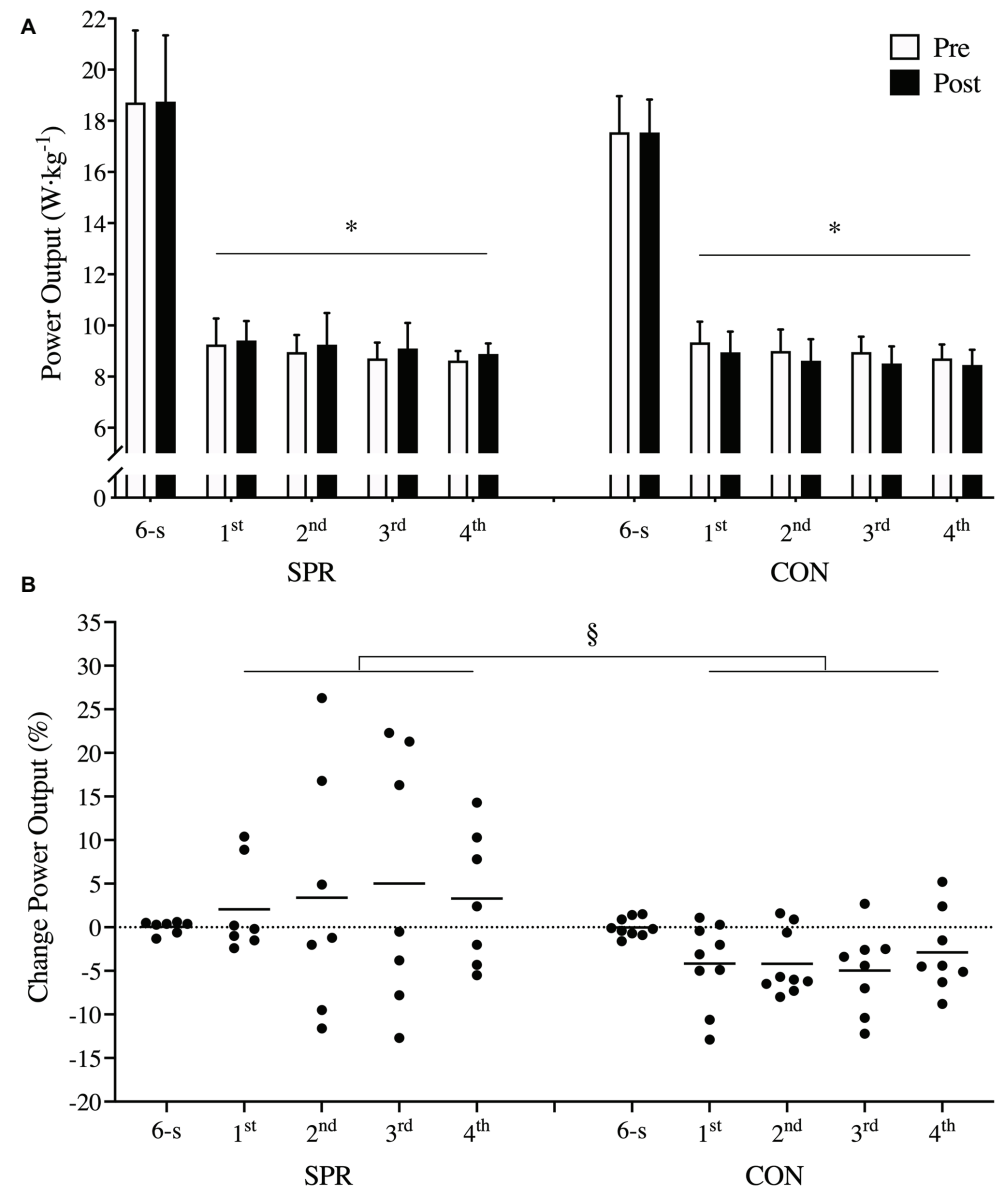
Peak power output (W·kg^−1^) on maximal 6-s sprint and mean power output on 4 × 30-s sprints (**A**, mean ± SD) before (Pre) and after (Post) a 3-week transition period of reduced training load in elite cyclists, including sprints in a low-intensity training session once a week [sprint group (SPR); *n* = 7] or only performing low-intensity training [control group (CON); *n* = 8] and individual percentage changes from Pre to Post **(B)**. ^*^indicates main effect of time (*p* < 0.05). ^§^indicates main effect of group on changes from Pre to Post (*p* < 0.05). Mean ± SD.

After the 3-week transition period, SPR had a larger increase in 30-s sprint performance than CON from Pre to Post (8 ± 11%, *p* = 0.01) with ES on changes between groups being moderate to large (ES: 0.6–1.7, Figure 3B). An overall, positive effect of time was observed in 30-s sprint performance in SPR (*p* = 0.04) and a negative effect of time was observed in CON (*p* = 0.01, Figure 3A). ES were considered small to moderate for all Post sprints in SPR (first sprint: 0.2 ± 0.3, second sprint: 0.4 ± 0.9, third sprint: 0.9 ± 1.1, fourth sprint: 0.7 ± 0.5) and small to moderate effects for CON (first sprint: −0.5 ± 0.3, second sprint: −0.5 ± 0.2, third sprint: −0.7 ± 0.3, fourth sprint: −0.5 ± 0.3) in relation to Pre. Peak power output during 6-s sprint did not change differently between groups (*p* = 0.59, Figure 3B) and did not change from Pre to Post in either group (Figure 3A).

### 20-min All-Out

**Figure 4:**
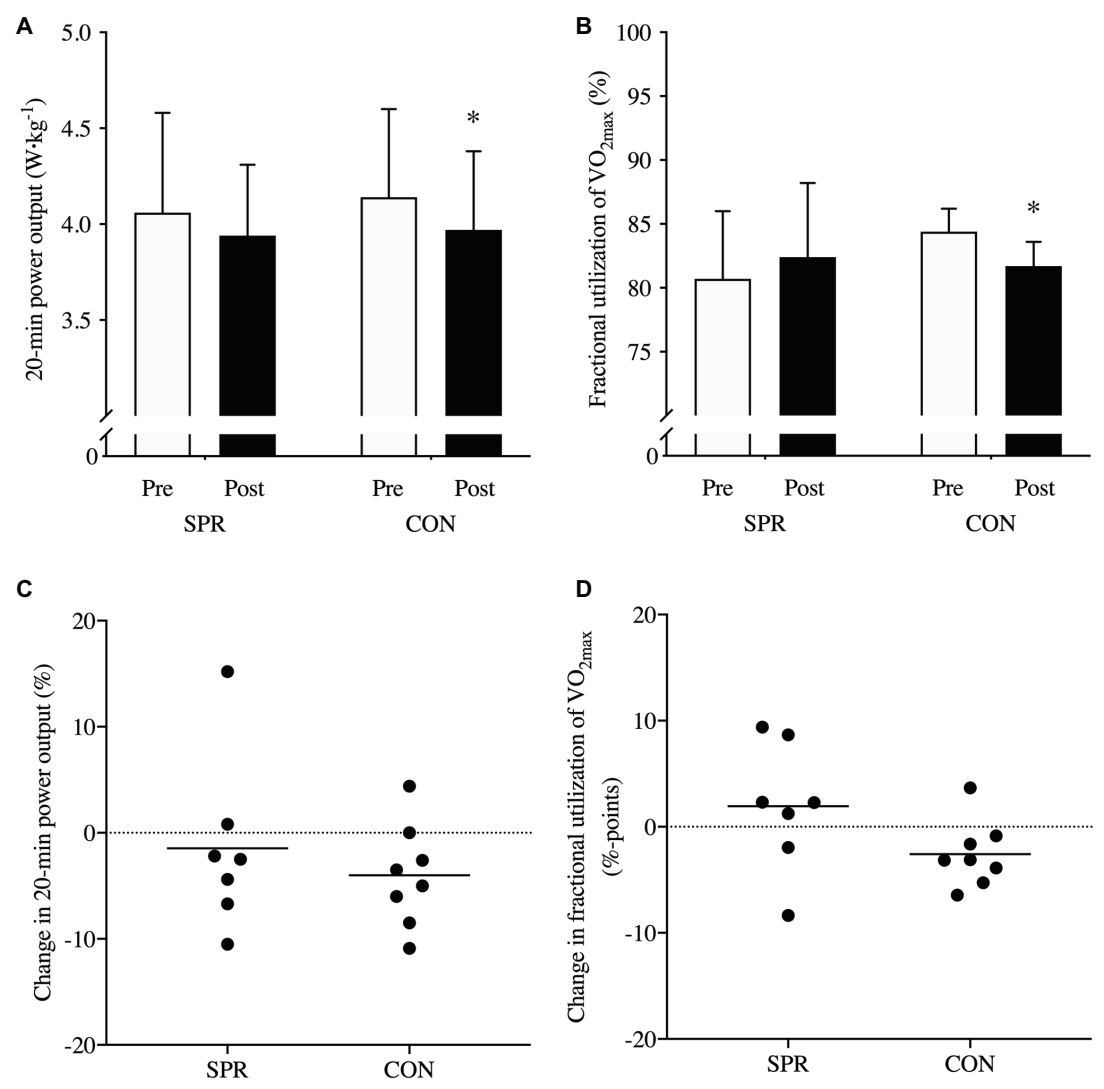
20-min all-out performance expressed in relative power output (W·kg^−1^; **A**) and percentage change **(C)** from before (Pre) to after (Post) a 3-week transition period of reduced training load in elite cyclists including sprints in a low-intensity training session once a week (SPR group; *n* = 7) or only performing low-intensity training (CON group; *n* = 8). Fractional utilization of VO_2max_ during 20-min all-out (%; **B**) and changes in %-points from Pre to Post **(D)**. ^*^indicates main effect of time (*p* < 0.05). Mean ± SD.

Twenty-minutes all-out performance did not change differently between groups (*p* = 0.63, ES: 0.1, Figure 4C). However, 20-min all-out performance was maintained from Pre to Post in SPR (−1 ± 5%, *p* = 0.37, ES: −0.2 ± 0.4), whereas a small decline of −3 ± 5% was observed in CON (*p* = 0.04, ES: −0.4 ± 0.3, Figure 4A). Fractional utilization of VO_2max_ during 20-min all-out did not change differently between groups but the difference in change was considered moderate (*p* = 0.19, ES: 0.8, Figure 4D). Specifically, SPR maintained utilization from Pre to Post (1.9 ± 6.1%-points, *p* = 0.18, ES: 0.2 ± 4.5), whereas CON decreased moderately (−2.5 ± 2.9%-points, *p* = 0.02, ES: −0.6 ± 2.3, Figure 4B). [BLa^−^] and RPE after 20-min all-out did not change differently between groups (*p* = 0.54 and *p* = 0.26, respectively) and was unaltered from Pre to Post in SPR and CON (Table 2). Likewise, the change in %HR_peak_ during 20-min all-out was not different between groups (*p* = 0.18) and was unaltered from Pre to Post in SPR and CON (Table 2). Stepwise multiple linear regression revealed that changes in fractional utilization of VO_2max_ during 20-min all-out (*p* < 0.01), VO_2max_ (*p* < 0.01), and GE in the semi-fatigued state (*p* = 0.05) explained the changes observed in 20-min all-out (*p* < 0.01, adjusted *R*^2^ = 0.89).

**Table 2:** Changes (Δ) in performance-related measures and body mass from before (Pre) to after (Post) a 3-week transition period of reduced training load in elite cyclists including sprints in a low-intensity training session once a week [sprint group (SPR); *n* = 7] or only performing low-intensity training [control group (CON); *n* = 9]. Mean ± SD.

	SPR			CON		
	Pre	Post	Δ	Pre	Post	Δ
[BLa^−^] 20-min all-out (mmol·L^−1^)	4.7 ± 3.3	5.6 ± 3.0	0.7 ± 2.2	7.0 ± 2.2	6.2 ± 1.6	−0.7 ± 2.2
RPE 20-min all-out	18.1 ± 2.9	18.1 ± 1.6	0.0 ± 2.8	18.9 ± 0.6	19.3 ± 1.1	0.4 ± 1.4
HR (% of HR_peak_)	91.8 ± 4.4	93.4 ± 1.3	1.6 ± 3.8	91.3 ± 0.9	92.3 ± 1.6	1.1 ± 1.6
GE fresh (%)	19.9 ± 1.0	19.5 ± 1.0	−0.4 ± 1.0	19.1 ± 1.0	19.2 ± 1.0	0.1 ± 1.0
GE semi-fatigued (%)	18.9 ± 1.0	18.9 ± 1.0	0.1 ± 1.0	19.1 ± 1.0	19.3 ± 1.0	−0.2 ± 1.1
ΔGE fresh vs. semi-fatigued	−1.0 ± 1.2^†^	−0.6 ± 1.1	−0.4 ± 1.2	0.0 ± 1.3	0.0 ± 1.1	0.0 ± 1.2
Body mass (kg)	73.6 ± 9.0	74.2 ± 9.4	0.7 ± 1.0	73.1 ± 4.8	73.7 ± 4.9^*^	0.8 ± 1.0
VO_2max_ (ml·min^−1^·kg^−1^)	73.4 ± 4.9	71.4 ± 4.0	−2.5 ± 5.7	71.3 ± 4.5	71.0 ± 4.8	−0.5 ± 4.0
W_max_ (W·kg^−1^)	6.0 ± 0.3	6.0 ± 0.3	1.1 ± 6.5	6.0 ± 0.5	6.0 ± 0.4	−0.9 ± 4.9

%HR_peak_, percent of peak heart rate during 20-min all-out; [BLa^−^], blood lactate concentration measured 1 min after conclusion of 20-min all-out; RPE, rate of perceived exertion immediately after 20-min all-out; GE, gross efficiency measured in steady-state periods in the fresh and the semi-fatigued state during the ~2.5 h long test protocol; ΔGE, change in gross efficiency from the fresh state to the semi-fatigued state (%-points); VO_2max_, maximal oxygen uptake; W_max_, maximal minute power output.

* indicates main effect of time (*p* < 0.05).

† significant difference between fresh and semi-fatigued state (*p* < 0.05).

### Performance-Related Measures and Body Mass

**Figure 5:**
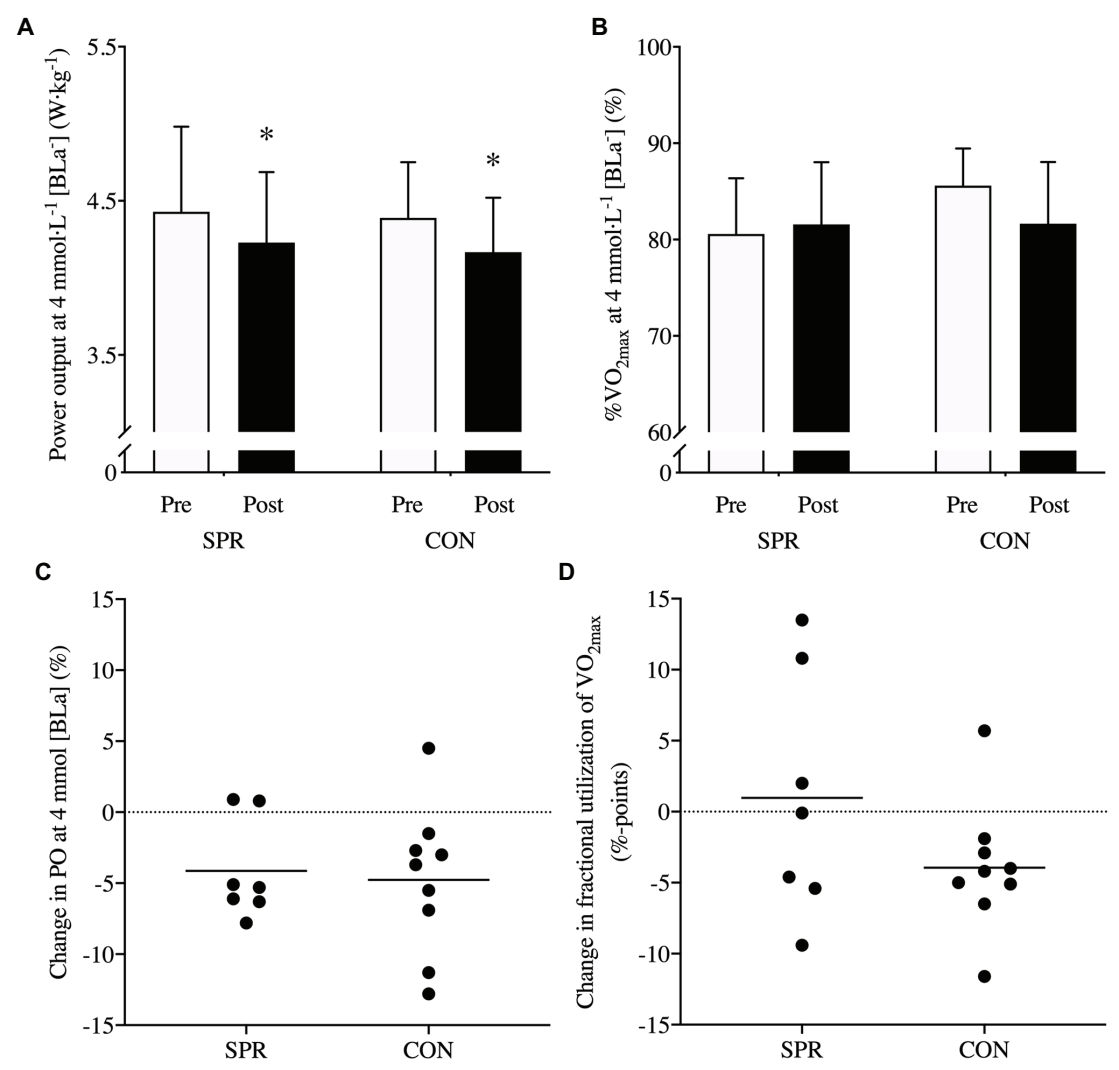
Relative power output at 4 mmol·L^−1^ [BLa^−^] W·kg^−1^ **(A)**, fractional utilization of VO_2max_ at 4 mmol·L^−1^ [BLa^−^] **(B)** and individual changes in percentage and %-points **(C,D)** from before (Pre) to after (Post) a 3-week transition period of reduced training load in elite cyclists including sprints in a low-intensity training session once a week (SPR group; *n* = 7) or only performing low-intensity training (CON group; *n* = 9). ^*^indicates main effect of time (*p* < 0.05). Mean ± SD.

Power output at 4 mmol·L^−1^ [BLa^−^] decreased similarly from Pre to Post (*p* = 0.83, ES: 0.1, Figure 5C) in SPR (−4 ± 4%, *p* = 0.02, ES: −0.4 ± 0.2) and CON (−5 ± 5%, *p* = 0.01, ES: −0.6 ± 0.4, Figure 5A). Fractional utilization of VO_2max_ at 4 mmol·L^−1^ [BLa^−^] did not change differently between groups but the ES was considered moderate (*p* = 0.16, ES: −1.0, Figure 5D). Specifically, SPR maintained fractional utilization of VO_2max_ at 4 mmol·L^−1^ [BLa^−^] (*p* = 0.69, ES: 0.2 ± 1.1) while CON approached significance to decrease moderately (*p* = 0.09, ES: −1.0 ± 0.7, Figure 5B). GE did not change differently between groups from Pre to Post in fresh (*p* = 0.18) or semi-fatigued state (*p* = 0.63; Table 3). The change in GE from fresh to semi-fatigued state was not different between groups at Pre (*p* = 0.13) or Post (*p* = 0.26); however, GE decreased from the fresh state to the semi-fatigued state in SPR at Pre (*p* = 0.02). The increase in body mass did not differ between groups from Pre to Post (*p* = 0.93, ES: 0.0, Table 2). Specifically, body mass tended to increase in SPR (*p* = 0.07, ES: 0.1 ± 0.1) and increased in CON from Pre to Post (*p* = 0.05, ES: 0.1 ± 0.1, Table 2). There was no difference in changes between groups in VO_2max_ or W_max_ and both groups remained unchanged from Pre to Post (Table 2).

**Table 3:** Strength parameters from before (Pre) to after (Post) a 3-week transition period of reduced training load in elite cyclists including sprints in a low-intensity training session once a week [sprint group (SPR); *n* = 7] or only performing low-intensity training [control group (CON); *n* = 9].

	SPR			CON		
	Pre	Post	Time	Pre	Post	Time
V_max_ (M·S^−1^)	4.0 ± 0.8	4.1 ± 0.9	*p* = 0.87	3.8 ± 08	4.2 ± 0.5	*p* = 0.02	
F_max_ (N)	3,030 ± 441	2,971 ± 528	*p* = 0.74	3,400 ± 902	3,095 ± 725	*p* = 0.11	
P_max_ (W)	1,516 ± 332	1,524 ± 460	*p* = 0.88	1,553 ± 251	1,611 ± 310	*p* = 0.25	

Effects of time are defined by *p*. Mean ± SD. V_max_, maximal velocity; F_max_, maximal force; P_max_, maximal power.

### Strength Parameters

Maximal velocity, F_max_ and P_max,_ did not change differently between groups (*p* = 0.13 *p* = 0.65, *p* = 0.36, respectively) and F_max_ and P_max,_ did not change within the group from Pre to Post (Table 3). However, V_max_ was increased by 14 ± 19% from Pre to Post in CON (*p* = 0.02).

### Burnout Symptoms

Total burnout did not change differently (*p* = 0.49) between SPR and CON and both groups were unchanged from Pre to Post. However, for the subscale “reduced sense of accomplishment,” the difference in development between groups approached significance (*p* = 0.09). In the change from “rarely” toward “sometimes, SPR did not change (Pre: 2.3 ± 0.5 vs. Post: 2.2 ± 0.5, *p* = 0.62) whereas CON approached significance” (Pre: 2.5 ± 0.7 vs. Post: 2.8 ± 0.5, *p* = 0.04). For “devaluation,” the difference in development between groups approached significance (*p* = 0.09) but neither SPR (Pre: 1.6 ± 0.6 vs. Post: 1.8 ± 0.7, *p* = 0.26) nor CON (Pre: 1.7 ± 0.4 vs. Post: 1.5 ± 0.4, *p* = 0.15) changed from Pre to Post. No group-differences or within-group changes were observed for “emotional and physical exhaustion.”

## Discussion

The present study investigated the effects of including 30-s sprints in a LIT-session once a week during a 3-week transition period of reduced training load in elite cyclists. The main finding was that inclusion of sprints in SPR improved sprint performance compared to LIT only in CON who had a deterioration hereof. Although no group differences occurred, 20-min all-out performance and fractional utilization of VO_2max_ during the 20-min test were maintained in SPR, whereas small to moderate declines were observed in CON. Power output at 4 mmol·L^−1^ [BLa^−^] was equally reduced in both groups, while VO_2max_, W_max_ and total burnout were unaffected in both groups.

### Sprint Performance

As expected, SPR improved 30-s sprint performance in the present study, whereas absence of sprinting led to deterioration of 30-s sprint performance in CON. Although sprint training has proven to be a potent training modality for both untrained and trained participants (Gist et al., 2014), this study is the first to show the potency of improving sprint performance in elite athletes even by inclusion of a relatively small amount of sprints (27 × 30-s) during the transition period. We also expected that an improved anaerobic capacity, indicated by the improved 30-s sprints, should improve short high-intensity endurance performance, such as W_max_ determined here. However, this was not the case in the present study, which is in contrast to previous studies where sprint training is added to the habitual volume of LIT (Laursen et al., 2002) or when sprints are implemented in LIT-sessions (Gunnarsson et al., 2019). This discrepancy could be related to the ~60% decrease in training load, the relatively short intervention of the present study, compared to previous studies (3 vs. 7–8 weeks) and smaller amounts of sprint training (27 vs. 96–144 × 30-s sprints; Laursen et al., 2002; Gunnarsson et al., 2019). In our approach, neither peak power during 6-s sprint nor V_max_ changed differently between groups but V_max_ was improved in CON only. This is in contrast to previous findings where improved peak power output (Fortes et al., 2019) and muscle strength (Martin et al., 1994) were found from short periods (2–4 weeks) of reduced training volume and maintained intensity-distribution in well-trained cyclists and runners. Inactivity has previously been reported to change fiber-type distribution toward type IIX phenotype (Coyle et al., 1985; Andersen and Aagaard, 2000). Hypothetically, an absence of type II muscle fiber activation as might be assumed during 3 weeks of LIT only and an absence of muscular activation might therefore favor a switch in fiber-specific characteristics, toward a more fast-twitch phenotype, possibly explaining an improved V_max_ in CON.

### 20-min All-Out Performance

Although changes in 20-min all-out performance did not differ between groups, performance was unaltered in SPR, whereas a small decline of ~3% was observed in CON. However, the relevance of avoiding a decrease in 20-min all-out performance after prolonged cycling would arguably be of importance in cycling competitions (van Erp et al., 2019). Endurance performance, such as 20-min all-out test, is mainly determined by fractional utilization of VO_2max_, VO_2max_, and efficiency and to a lesser fraction anaerobic capacity (Jeukendrup et al., 2000; Joyner and Coyle, 2008). In the current study, the maintained 20-min performance in SPR was coincided by maintained fractional utilization of VO_2max_ during the test, whereas it decreased by ~3%-points in CON. However, VO_2max_, W_max_, or GE in fresh state or semi-fatigued state did not change in any group, and both SPR and CON showed similar decreases in power output at 4 mmol·L^−1^ [BLa^−^]. Thus, the different development pattern in fractional utilization of VO_2max_ within groups is probably the main explanation for the changes in 20-min all-out performance. This was further confirmed by a stepwise multiple linear regression analysis, where changes in fractional utilization of VO_2max_ during 20-min all-out, together with changes in VO_2max_ and GE explained 89% of the variance in 20-min all-out changes, supporting the importance of these variables for high-intensity endurance performances (Jeukendrup et al., 2000; Joyner and Coyle, 2008).

The reductions in submaximal exercise measures in CON, such as fractional utilization of VO_2max_ are possibly related to a decreased oxidative capacity (Coyle et al., 1984), which has been reviewed to decline with training reduction in a volume-dependent fashion (Neufer, 1989). Maintaining or increasing intensity of exercise during such reduced training volumes, however, seems of importance to maintain submaximal endurance performance (Neufer, 1989; Ronnestad et al., 2014), probably explaining the unchanged fractional utilizations of VO_2max_ in SPR. This is supported by a previous study of inclusion of sprint training in a 4-week period of 65% decreased training volume, where mitochondrial oxidative enzyme activity was maintained (Iaia et al., 2009). Furthermore, the present study and others (Neufer, 1989; Rietjens et al., 2001) show that maintaining 30–50% of the training volume maintains VO_2max_ in trained and elite cyclists for short periods (3 weeks). The importance of maintaining a minimum of endurance training is showed in studies where training cessation decreases VO_2max_ by 7–11% after 3–5 weeks in trained athletes (Coyle et al., 1984; Maldonado-Martin et al., 2017). Changes in blood volume and hemoglobin mass are regarded as main causes for changes in VO_2max_ (Coyle et al., 1986), and the unchanged VO_2max_ in the present study is supported by an unchanged blood volume and hemoglobin mass, although this measure was only performed on a sub-set of the participants (see Appendix). However, small decreases <200 ml in blood volume has recently been shown not to alter VO_2max_ (Skattebo et al., 2020), which could have been the case in our short intervention study. Overall, our study indicates that elite cyclists are able to reduce training load by ~60% for short periods without affecting the maximal aerobic power.

### Mental Recovery

One might expect a decrease in the burnout markers during the transition period due to the great reduction in training load and absence of strenuous competitions, which has earlier been argued a necessity for elite athletes (Mujika et al., 2018). However, in our study, total burnout was unchanged from Pre to Post within both groups. The average score for all subscales were comparable to a recent study in a population of young elite-sportsmen (Gerber et al., 2018), and the general low scores in the mental subscales indicates a state of relatively low burnout in the elite cyclists, possibly explaining why this does not change during a 3-week transition period. In addition, only small changes were observed in the subscales, which indicates that changes in mental recovery might be difficult to measure during such short periods in a small group of elite cyclists. In any case, inclusion of sprints in one weekly LIT-session during the transition period does not seem to pose any effect on mental recovery compared to LIT only in elite cyclists with initially low levels of burnout scores.

### Limitations

The relatively short intervention applied in the current study yields limited insight into the effects of including sprints in LIT-sessions on performance and mental recovery in elite cyclists. The lack of control of the training and competitions performed prior to the intervention might affect the outcomes, despite our effort for matching the groups according to training load and fitness. In addition, food consumption was not strictly controlled for in the present study and might have introduced unaccounted noise in the outcomes. However, with the unchanged body composition (see Appendix) and well-developed nutritional routines among elite athletes, we regard this possible effect to be small. After the short transition periods of typically 2–3 weeks, elite cyclists often increase training load gradually. Whether the current small, positive effects observed in SPR compared to CON translate into improved performance later in the preparatory period and competition period, however, needs further investigation.

In conclusion, including series of 30-s sprints in a LIT-session once a week during a 3-week transition period improves sprint performance compared to LIT only. In addition, 20-min all-out performance and fractional utilization of VO_2max_ was maintained in SPR while LIT only reduced these variables. Inclusion of sprints does not affect the power output at 4 mmol·L^−1^ [BLa^−^], which was equally reduced in both groups. However, neither VO_2max_ and W_max_ nor total burnout seem affected by a 3-week transition period with severely reduced training load independent of sprinting. Inclusion of sprints in LIT-sessions may therefore be a plausible, time-efficient strategy to maintain performance for elite cyclist during short periods of reduced training load without affecting mental recovery.

## Data Availability Statement

The raw data supporting the conclusions of this article will be made available by the authors, without undue reservation.

## Ethics Statement

The studies involving human participants were reviewed and approved by Local Ethical Committee at Inland Norway University of Applied Sciences. The patients/participants provided their written informed consent to participate in this study.

## Author Contributions

NA, MS, MK, KS, ØS, and BR contributed to conception and design of the study. NA, IL, PB, MK, and KS executed the study and collected data. NA performed the statistical analysis. NA wrote the first draft of the manuscript. All authors contributed to manuscript revision, read, and approved the submitted version.

### Conflict of Interest

The authors declare that the research was conducted in the absence of any commercial or financial relationships that could be construed as a potential conflict of interest.
