# Supplementary material for: Effects of Including Sprints in One Weekly Low-Intensity Training Session During the Transition Period of Elite Cyclists
Source: Front Physiol. 2020 Sep 11;11:1000. doi: 10.3389/fphys.2020.01000 (PMC7518025; doi:10.3389/fphys.2020.01000)
Supplement: Supplementary file 1 [file Data_Sheet_1.docx]

Appendix

*Table 4: Hematological measures and body composition before (Pre) and after (Post) a 3-wk transition period of reduced training load in elite cyclists including sprints in a low-intensity training session once a week (SPR, Sprint group, n=7) or only performing low-intensity training (CON, Control group, n=9). Mean ± SD. Hematological measures were determined using a modified version of the carbon monoxide (CO) rebreathing technique using an OpCO (WGT, Austria) and venous blood samples, using pre-heparinized syringes (PICO50 80IU, Radiometer, DK) were analyzed in triplicate for carboxy-Hb (%HbCO) on a hemoximeter (ABL800, Radiometer, Copenhagen, Denmark). Body composition was measured after an overnight fast using a dual-energy x-ray absorptiometry (DXA) scan on a Lunar Prodigy (GE-Lunar Prodigy, Madison, WI, USA, EnCore software version 15). CO rebreathing and DXA-scan was unfortunately only possible to performed at two and three of the centers, respectively, hence a low n is reported for these measures.*

|  | SPR | | |  | CON | | |  |
| --- | --- | --- | --- | --- | --- | --- | --- | --- |
|  | Pre | Post | Time |  | Pre | Post | Time | Group |
|  | *Hematological measures (sprint n=4, control n=5)* | | | | | | |  |
| Hb-mass (g·kg^-1^) | 13.2 ± 0.5 | 13.0 ± 0.5 | p= .47 |  | 13.8 ± 1.4 | 13.6 ± 1.2 | p= .37 | p= .40 |
| BV (mL) | 6355 ± 630 | 6479 ± 648 | p= .41 |  | 7246 ± 485 | 7209 ± 689 | p= .32 | p= .10 |
| PV (mL) | 3709 ± 354 | 3828 ± 355 | p= .31 |  | 4202 ± 253 | 4082 ± 377 | p= .26 | p= .12 |
| RBCV (mL) | 2646 ± 284 | 2651 ± 316 | p= .92 |  | 3044 ± 398 | 3027 ± 386 | p= .70 | p= .15 |
| *Body composition (sprint n=4, control n=6)* | | | | | | | | |
| LBM (kg) | 63.0 ± 6.1 | 61.6 ± 6.9 | p= .29 |  | 63.6 ± 6.1 | 60.3 ± 5.7 | p= .01 | p= .92 |
| Body fat (%) | 11.0 ± 1.5 | 11.6 ± 1.4 | p= .27 |  | 12.1 ± 5.3 | 12.6 ± 5.9 | p= .29 | p= .73 |

*Hemoglobin mass (Hb-mass), blood volume (BV), plasma volume (PV), red blood cell volume (RBCV), lean body mass (kg) and body fat.*
